# Response to antiretroviral therapy (ART): comparing women with previous use of zidovudine monotherapy (ZDVm) in pregnancy with ART naïve women

**DOI:** 10.1186/1471-2334-14-127

**Published:** 2014-03-04

**Authors:** Susie Huntington, Claire Thorne, Jane Anderson, Marie-Louise Newell, Graham P Taylor, Deenan Pillay, Teresa Hill, Pat Tookey, Caroline Sabin

**Affiliations:** 1 Centre for Paediatric Epidemiology and Biostatistics, UCL Institute of Child Health, 30 Guilford Street, London WC1N 1EH, UK; 2 HIV Epidemiology and Biostatistics Group, UCL Research Department of Infection and Population Health, Royal Free Campus, Rowland Hill Street, London NW3 2PF, UK; 3 Homerton University Hospital NHS Foundation Trust, Homerton Row, London E9 6SR, UK; 4 Faculty of Medicine, University of Southampton, Building 85, Life Sciences Building, Highfield Campus, University Rd, Southampton SO17 1BJ, UK; 5 Faculty of Medicine, Imperial College, London, UK; 6 Public Health England (PHE) CfI, 61 Colindale Avenue, London NW9 5HT, UK

**Keywords:** HIV, Pregnancy, Antiretroviral therapy, United Kingdom

## Abstract

**Background:**

Short-term zidovudine monotherapy (ZDVm) remains an option for some pregnant HIV-positive women not requiring treatment for their own health but may affect treatment responses once antiretroviral therapy (ART) is subsequently started.

**Methods:**

Data were obtained by linking two UK studies: the UK Collaborative HIV Cohort (UK CHIC) study and the National Study of HIV in Pregnancy and Childhood (NSHPC). Treatment responses were assessed for 2028 women initiating ART at least one year after HIV-diagnosis. Outcomes were compared using logistic regression, proportional hazards regression or linear regression.

**Results:**

In adjusted analyses, ART-naïve (n = 1937) and ZDVm-experienced (n = 91) women had similar increases in CD4 count and a similar proportion achieving virological suppression; both groups had a low risk of AIDS.

**Conclusions:**

In this setting, antenatal ZDVm exposure did not adversely impact on outcomes once ART was initiated for the woman’s health.

## Background

In the UK, zidovudine monotherapy (ZDVm) has been widely used for prevention of mother-to-child-transmission (PMTCT). Although combination antiretroviral therapy (ART) is now more commonly used for this purpose, ZDVm remains an option for pregnant women not on therapeutic ART with high CD4 counts (>350 cells/mm^3^) [1], low viral loads (<10,000 copies/ml), and who are willing to deliver by elective caesarean section [2]. The 2012 BHIVA guidelines recommend that women opting to use ZDVm for PMTCT start ZDVm before 24 weeks of pregnancy [2]. Pregnant women not on ART with CD4 ≤ 350 cells/mm^3^ are recommended to initiate long-term ART, as per the general UK HIV treatment guidelines [3].

Little is known about the impact of short-term ZDVm exposure on the woman’s subsequent response to ART when started for her own health. In low- and middle-income settings use of single-dose nevirapine (sd-NVP) can have a negative impact on subsequent treatment responses to NVP-containing regimens, with high levels of drug resistance, particularly when ART is initiated within 6–12 months post-sd-NVP exposure [4,5]. However, whereas resistance to NVP requires a single mutation, resistance to ZDV requires multiple sequential mutations. As such, the development of resistance following short-term ZDVm for PMTCT is uncommon [6-9] and limited to women with more advanced disease [10-13] who would not meet clinical criteria for ZDVm PMTCT use [2]. In contrast to the UK guidelines, ZDVm for PMTCT is no longer recommended within the updated consolidated World Health Organization (WHO) guidelines on use of ART; in line with the WHO guidelines’ main focus on earlier initiation of ART to decrease transmission, pregnant women not yet on therapy are recommended to start long-term combination therapy regardless of CD4 count [14]. Despite these recent changes, there are many women in low- and middle-income settings with previous antenatal exposure to ZDVm who have yet to start ART for their own health [15]. In addition, use of ZDVm in pregnancy will remain a strategy for PMTCT in some settings until combination ART becomes more accessible.

Our aim was to test whether short-term exposure to ZDVm in a previous pregnancy has an adverse effect on treatment outcomes once a woman starts ART for her own health. Record linkage between the UK and Ireland National Study of HIV in Pregnancy and Childhood (NSHPC) and the UK Collaborative HIV Cohort (UK CHIC) study gave us the opportunity to address this issue, which has implications for women with previous ZDVm experience.

## Methods

We compared treatment outcomes among ART-naïve and ZDVm-experienced women starting therapeutic ART for their own health. Data were obtained from the UK CHIC study, an observational cohort that collates clinical data for adults receiving HIV-care at 15 large HIV clinics [16], and the NSHPC which collects antenatal data on all pregnant women diagnosed HIV-positive in the UK and Ireland [17]. Women reported to both studies were linked using demographic and clinical variables, as described elsewhere [18]. Data were not available on whether women had pregnancies prior to HIV diagnosis or infection, nor were data available on previous ART use outside the UK. Eligibility criteria were: initiating therapeutic ART at a UK CHIC site in 2000–2009 at least one year after HIV-diagnosis, either ZDVm-experienced or ART-naïve (combination ART-experienced women were excluded), and aged ≤49 years at HIV-diagnosis. Women were categorised as ZDVm-experienced if, according to NSHPC or UK CHIC data, they had ever used short-term ZDVm during pregnancy.

Baseline CD4 count and viral load were taken as the latest measurement within the three months before ART initiation. Characteristics of women starting treatment were compared using the Chi-square, Fisher’s exact or Wilcoxon two-sample test. ART outcomes were compared using logistic regression, proportional hazards regression or linear regression.

The UK CHIC Study has multicentre ethics committee approval (MREC/00/7/47). Ethics approval for NSHPC was renewed following review by the London Multi-Centre Research Ethics Committee in 2004 (MREC/04/2/009).

## Results

Overall, 1937 ART-naïve and 91 ZDVm-experienced women started therapeutic ART in 2000–2009. ZDVm-experienced women had used ZDVm in either one (n = 84) or two pregnancies (n = 7). No infants acquired HIV. ZDVm was used for a median of 12 weeks (IQR 8–16) and was typically started at 28 weeks gestation (range 17–39, IQR 24–31). The median duration between delivery (of the latest pregnancy) and starting therapy was 43 months (IQR 30–63); six women started within 12 months of delivery, none within 6 months.

In both groups some women were known to have had one or more previous pregnancies during which no ART was used (2.6% (51/1937) ART-naïve women and 11.0% (10/91) ZDV-experienced women). These pregnancies either ended early, due to termination or miscarriage, or resulted in a live birth where HIV was not diagnosed until delivery.

The baseline demographic and clinical characteristics of ZDVm-experienced and ART-naïve women at the time of starting therapeutic ART are summarised in Table 1. The median follow-up time was the same for both groups (4 [IQR 2–6] years, p = 0.77). Median time since HIV-diagnosis was 5 [4-9] years for ZDVm-experienced and 4 [2-7] years for ART-naïve women (p < 0.001). ZDVm-experienced women were younger than ART-naïve women (33 [30–37] and 35 [30–40] years, respectively, p = 0.01), more likely to have been infected heterosexually (97.8% vs. 88.7%, p = 0.006) and more likely to start therapy during pregnancy (29.7% vs. 7.6%, p = 0.001). A similar proportion of women were of black ethnicity (black-African, black-Caribbean or black-other) (ZDVm-experienced: 73.6%; ART-naïve: 69.6%, p = 0.42). Overall, 28.3% (n = 574) used a PI-based regimen (ritonavir-boosted or non-boosted), 61.6% (n = 1249) an NNRTI-based regimen and 10.1% (n = 205) an NRTI or other regimen. The regimens used were similar regardless of prior ZDVm experience (p = 0.48). ZDVm-experienced women were more likely to have at least one viral load measurement recorded in the first year of treatment (ZDVm-experienced: 95.6%; ART-naïve: 85.9%, adjusted Odds Ratio 3.24 [95% confidence interval 1.08-9.75], p = 0.04), however the median number of measurements recorded was the same (ZDVm-experienced: median 4 [IQR 3–5]; ART-naïve: median 4 [2-5], p = 0.83).

**Table 1:** Characteristics of ART-naïve and ZDVm-experienced women when starting therapeutic ART in 2000-2009

**Demographic and clinical characteristics at start of therapeutic ART**		**ZDVm-experienced (n = 91)**		**ART-naïve (n = 1937)**		**p-value**
Age (years), median (IQR)		33	(30–37)	35	(30–40)	0.01
Time since HIV diagnosis (years), median (IQR)		5	(4–9)	4	(2–7)	<0.001
Pregnant, n (%)		27	(29.7)	147	(7.6)	<0.001
Ethnicity, n (%)	Black	67	(73.6)	1349	(69.6)	0.42
Non-black/not known		24	(26.4)	588	(30.3)	
Risk group, n (%)	Heterosexual sex	89	(97.8)	1718	(88.7)	0.006
Other		2	(2.2)	219	(11.3)	
Year, n (%)	2000-2002	13	(14.3)	375	(19.4)	0.40
	2003-2005	32	(35.1)	589	(30.4)	
	2006-2009	46	(50.6)	973	(50.2)	
ART regimen started, n (%)						
PI based (boosted and non-boosted)		22	(24.2)	552	(28.5)	0.48
NNRTI		57	(62.6)	1192	(61.5)	
NRTI/other		12	(13.2)	193	(10.0)	
Baseline CD4 count	Median (IQR), (cells/mm^3^)	226	(162–339)	225	(150–304)	0.16
(n = 75, n = 1431)	CD4 <200 cells/mm^3^, n (%)	29	(38.7)	584	(40.8)	0.71
	CD4 <350 cells/mm^3^, n (%)	57	(76.0)	1195	(83.5)	0.09
Baseline viral load	Median (IQR), (log_10_ copies/ml)	4.1	(3.2-4.5)	4.3	(3.4-4.9)	0.08
(n = 68, n = 1371)	≤50 copies/ml, n (%)	4	(5.9)	126	(9.2)	0.35
	≤400 copies/ml, n (%)	8	(11.8)	228	(16.6)	0.29
	≤10,000 copies/ml, n (%)	28	(41.2)	535	(39.0)	0.72
Hepatitis C co-infection, n (%)		4	(4.4)	170	(8.8)	0.14
Hepatitis B co-infection, n (%)		1	(1.1)	55	(2.8)	0.32
Previous AIDS event, n (%)		7	(7.7)	279	(14.4)	0.07

IQR, Interquartile range; PI, protease inhibitor; NNRTI, non-nucleoside reverse transcriptase inhibitor; NRTI, nucleoside reverse transcriptase inhibitor.

ZDVm-experienced and ART-naïve women started therapeutic ART at similar baseline CD4 counts (ZDVm-experienced: 226 [162–339] cells/mm^3^; ART-naïve: 225 [150–302] cells/mm^3^, p = 0.16) and viral load (ZDVm-experienced: 4.1 [3.2-4.5] log10 copies/ml; ART-naïve: 4.3 [3.4-4.9] log10 copies/ml, p = 0.08). Few women in either group were known to have hepatitis B (ZDVm-experienced: 1.1%; ART-naïve: 2.8%, p = 0.32) or hepatitis C co-infection (ZDVm-experienced: 4.4%; ART-naïve: 8.8%, p = 0.14). Few women had previously had an AIDS event (ZDVm-experienced: 7.7%; ART-naïve: 14.4%, p = 0.07).

ZDVm-experienced and ART-naïve women had similar treatment outcomes (risk of an AIDS event or death, CD4 cell count change) in the first year of therapy (Table 2). Where viral load data were available, most women had undetectable viral load at 12 months (77.9%, 1160/1490). ZDVm-experienced women were more likely to achieve virological suppression (≤50 copies/ml) within the first year of treatment (Table 2) and achieved virological suppression more quickly than ART-naïve women (median 2.5 [IQR 1.3-3.4] months versus 3.0 [1.7-4.8] months, respectively, hazard ratio (HR): 1.30 [95% CI 1.03-1.64], p = 0.03, aHR: 1.28 [1.01-1.62], p = 0.04).

**Table 2:** Treatment outcomes for ART-naïve and ZDVm-experienced women starting therapeutic ART in 2000-2009

**Variable**	**ZDVm-experienced N = 91**		**ART-naïve N = 1937**		Unadjusted/Adjusted*	**95% CI**	**p-value**
Death/AIDS event within 1 year, n (%)	1	(1.1)	92	(4.8)	0.45	0.16-1.19	0.11
					0.59	0.22-1.60	0.30
CD4 cell count change at 6 months, median cells/mm^3^ (IQR)^a^	106	(41–171)	106	(34–197)	−0.66	−49.5-24.6	0.51
					−0.68	−44.6-29.2	0.68
CD4 cell count change at 12 months, median cells/mm^3^ (IQR)^b^	153	(61–233)	160	(70–256)	−1.2	−71.3-18.1	0.24
					−0.83	−63.3-25.7	0.41
Virological suppression at 6 months, n (%)^c^	53	(74.7)	1115	(74.4)	1.01	0.59-1.75	0.96
					1.00	0.56-1.73	0.97
Virological suppression at 12 months, n (%)^d^	52	(78.8)	1108	(77.8)	1.06	0.58-1.94	0.85
					1.06	0.57-1.96	0.86
Achieved virological suppression within 1 year, n (%)^e^	75	(86.2)	1408	(84.7)	1.30	1.03-1.64	0.03
					1.28	1.01-1.62	0.04
Virological rebound among those achieving virological suppression within 6 months, n (%)^f^	16	(22.9)	197	(16.6)	1.54	0.93-2.57	0.10
					1.51	0.90-2.53	0.12

^a^ZDVm: n = 59 and ART-naïve: n = 1272; ^b^ZDVm: n = 58 and ART-naïve: n = 1192; ^c^ZDVm: n = 71 and ART-naïve: n = 1499; ^d^ZDVm: n = 66 and ART-naïve: n = 1424; ^e^ZDVm: n = 70 and ART-naïve: n = 1189; ^f^ZDVm: n = 70 and ART-naïve: n = 1189.

Estimates are odds ratios (viral suppression at 6 and 12 months), hazard ratios (death/AIDS event, virological suppression within 1 year, virological rebound) or difference in medians (CD4 cell count change at 6 and 12 months).

*Variables adjusted for are: age at start of ART, exposure group, ethnicity, time since HIV-diagnosis, year of starting ART, previous AIDS event, baseline viral load category, baseline CD4 count category and hepatitis B/C co-infection.

## Discussion

This UK study indicates that where ZDVm is used in pregnancy to prevent MTCT among women with high CD4 count and viral load <10,000 copies/ml it does not have a deleterious effect on treatment outcomes when ART is subsequently started. This adds support to the limited number of studies which indicate that short-term use of ZDVm for PMTCT is not detrimental to women’s long-term health [7,9,19,20] and provides some reassurance with respect to the large number of women in lower-resourced settings with prior antenatal ZDVm exposure who have not yet initiated treatment. However, as a substantial proportion of these women may have had higher viral load in pregnancy [21,22], their outcomes may be different. The increased likelihood of achieving viral suppression among ZDV-experienced women may be due to better treatment adherence or frequency of viral load monitoring. ZDVm-experienced women were more likely to have a viral load measure reported in the first year of treatment indicating that they had better contact with clinical care. If having a previous pregnancy, and short-term use of ART in that pregnancy, results in better engagement in clinical care when a woman subsequently starts therapy for her own health, this could mask any deleterious effect of the previous ART exposure. No data were available on previous pregnancies before HIV diagnosis or ART use outside the UK, something that may impact treatment outcomes. Therefore, further investigation is required to assess the long-term impact of short-term antenatal ART used for PMTCT.

## Conclusions

In this setting, antenatal ZDVm exposure did not adversely impact on outcomes once ART was initiated for the woman’s health. This was a small study with limited statistical power and further research is required to support these findings.

## Competing interests

The authors declare that they have no competing interests.

## Authors’ contributions

SH carried out the statistical analysis and drafted the manuscript. TH undertook data acquisition. All authors contributed to the interpretation of data and drafting of the manuscript. All authors read and approved the final manuscript.

## Pre-publication history

The pre-publication history for this paper can be accessed here:

http://www.biomedcentral.com/1471-2334/14/127/prepub
